# Biotremological research using a DIY piezoelectric contact microphone - examples with insects

**DOI:** 10.3897/BDJ.13.e143481

**Published:** 2025-02-14

**Authors:** Ilia Gjonov, Albena Lapeva-Gjonova, Monika Pramatarova

**Affiliations:** 1 Sofia University, Faculty of Biology, Sofia, Bulgaria Sofia University, Faculty of Biology Sofia Bulgaria; 2 National Museum of Natural History - Bulgarian Academy of Sciences, Sofia, Bulgaria National Museum of Natural History - Bulgarian Academy of Sciences Sofia Bulgaria

**Keywords:** biotremology, Hemiptera, Hymenoptera, Coleoptera, vibroacoustics

## Abstract

This study presents a new design of sensor tool to record substrate-borne vibrations produced by insects. We applied a piezo element acting as a contact microphone connected to a digital recorder to detect the signals emitted by insects. A suitable 3D printed microphone box with a mechanism of connection to the substrate or to soft tweezers holding the insect is created. We found that the recordings of the low-frequency signals (up to 20 кHz) were sufficiently good for analysis and, at the same time, a much faster and easier method than the common ones of detecting micro-vibrations using a piezoelectric sensor and, importantly, is incomparably cheaper than using a laser vibrometer. This setup is suitable for the detection and structural description of signals emitted by insects and other arthropods. Oscillograms, spectrograms and audio files of the recorded signals of selected ants (*Manicarubida*, Latreille, 1802, *Messorwasmanni* Krausse, 1910, *Myrmicaravasinii* Finzi, 1923) and *Poneracoarctata* (Latreille, 1802)), an ant nest beetle (*Paussusturcicus* I. Frivaldszky von Frivald, 1835), a planthopper (*Orosangajaponica* (Melichar, 1898)) and a jumping plant louse (*Bactericeraperrisii* Puton, 1876) are provided to demonstrate the effectiveness of the created equipment. The recordings from stridulation in *Myrmicaravasinii*, *Manicarubida*, *Poneracoarctata* and *Paussusturcicus* and the male call song of *Orosangajaponica* represent the very first documented signal production for these species. A scheme of the contact microphone and its mode of connection is shown. The research presented will democratise biotremological methods for the needs of integrative taxonomy and behavioural ecology, providing a broader understanding of vibrational signals through an efficient, accessible and operational method for both professional and citizen scientists.

## Introduction

Biotremology is a new, rapidly growing discipline that studies communication by surface-borne vibrations detected by specialised sensory receptors and organs in animals (Hill and Wessel 2016, Hill et al. 2022, Roberts and Wickings 2022). Unlike the acoustic signal (sound), which is transmitted in a uniform fluid medium (gas, liquid) by longitudinal compression waves, vibration signals are transmitted in a solid environment (e.g. plant, soil) by mechanical waves with low speed and frequency of 50-5000 Hz (Michelsen et al. 1982, Hill and Wessel 2016). Vibrations and sound waves, commonly referred to as vibroacoustics, cannot be easily separated since the same physical mechanism usually generates both simultaneously (Masoni et al. 2021).

At least 200,000 species rely on vibrational communication, many of them exclusively (Cocroft and Rodríguez 2005, Hill and Wessel 2016, Virant-Doberlet et al. 2023). This ancient form of communication has been documented in 17 insect orders and 148 families, with a preponderance of studies in Hemiptera, Hymenoptera and Coleoptera (Turchen 2021). Although there are different taxon-specific names for the behavioural mechanisms used by animals to send vibrational signals, they refer to drumming, stridulation, tremulation, tymbal and vocalisation (Hill and Wessel 2016). In essence, vibrations are an important communication and sensory tool for insects, often associated with mating, territorial display, alarm, predator avoidance and detection, foraging and coordination in social insects (Virant-Doberlet and Čokl 2004, Čokl and Virant-Doberlet 2009).

Records of vibroacoustic signalling still represent only a small fraction of the presumably huge percentage of insects in which such communication is thought to occur (Cocroft and Rodríguez 2005). For example, there are records for only 40 species of bark beetles (Curculionidae, Scolytinae) out of 6000 (Arjomandi et al. 2024) and comparable knowledge of acoustic communication in jumping plant lice, with just over 100 species out of 4000 described so far (Liao et al. 2022). There is a huge gap in the available records of vibroacoustic communication in one of the dominant groups of terrestrial insects, such as ants. This is even more true for Palaearctic species, despite their comparatively good taxonomic investigation. For the latter, signal data are available for a limited number of species in the genera *Camponotus*, *Crematogaster*, *Myrmica*, *Messor*, *Leptothorax* and *Pheidole* (Fuchs 1976, Zhantiev and Sulkanov 1977, Stuart and Bell 1980, Grasso et al. 2000, Barbero et al. 2009, Sala et al. 2014, Di Giulio et al. 2015, Casacci et al. 2021, Masoni et al. 2021). Furthermore, using vibroacoustic communication to discover cryptic ant species highlights its potential for integrative taxonomy (Ferreira et al. 2010, Peña Carrillo et al. 2021).

Several techniques and types of sensors have been developed to accurately detect the substrate-borne signals produced by insects, depending on the specific requirements of the research. The most common are laser Doppler vibrometry (LDV), piezoelectric accelerometers, gramophone cartridges and micro-electromechanical sensors (MEMS).

**LDV** is one of the most widely used vibration recording techniques and is considered the 'gold standard' for recording (Hill et al. 2022). It uses laser beams to measure the velocity of surface vibrations without physical contact, making it highly precise and non-invasive. LDVs can record vibrations at a very small point, providing opportunities for spatial signal propagation studies. Particularly rich are the capabilities of scanning LDVs, which can also record 4D sound (taking into account spatial and temporal metrics). A disadvantage of these sensors is their very high cost, which makes them inaccessible in cases where acoustic research is carried out by hobbyists or by scientists whose main work is not biotremology. Due to their high precision, LDVs are well suited for use in laboratory conditions, but have some disadvantages related to their high mass, high power consumption and overheating in warm environments (Šturm et al. 2019).

**Piezoelectric accelerometers** convert mechanical vibrations into electrical signals, which can then be recorded and analysed using materials that generate a voltage when subjected to mechanical stress (the piezoelectric effect). These sensors are widely used for recording insect microvibrations, particularly in plants, as they can be attached to stems or leaves to measure substrate-borne vibrations. Piezoelectric accelerometers have been used to detect mating calls, territorial signals and alarm signals in various insect species (e.g. Stuart and Bell (1980), Cocroft et al. (2000), Wenninger et al. (2009)).

**Grammophone cartridges** can be of two main types: dynamic (inductive) or piezoelectric. They are widely used as sensors in biotremological research (Cocroft et al. 2000, Tishechkin 2007, Percy et al. 2008, Liao and Yang 2015, Lin et al. 2019, Tishechkin 2023). They are suitable for recording sound both in the laboratory and in the field. They are low-noise, sensitive and relatively inexpensive. Their main disadvantage is that they are relatively complicated to use, requiring the substrate to be pressed against the cartridge needle with a precise force.

**MEMS** are miniature devices that incorporate mechanical and electronic components on a micro-scale. Although they are based on the piezo effect, changes in capacitive or inductive reactance or other physical phenomena similar to microphones, their common feature is miniaturisation. These sensors are increasingly being used in insect vibration studies due to their compact size, sensitivity and versatility. MEMS can detect very small amplitude vibrations and are often used in portable recording setups. MEMS-based sensors were used to study the role of microvibrations in honeybee hives (Nolasco et al. 2019). This type of sensor is used relatively infrequently and its potential for biotremological research is likely to be exploited in the future.

The choice of sensor and technique depends on the specific research question, insect species and substrate involved. Each method has its strengths and limitations, making it important to select the appropriate tool based on the study requirements. Researchers often combine these methods to obtain a comprehensive picture of insect vibrational communication.

In the search for a low-cost method for detecting insect vibrations, we have designed a new DIY piezoelectric contact microphone as an accessible and efficient tool that can be used in a variety of settings. It could enable researchers, particularly those in resource-limited environments, to carry out sophisticated vibration studies without the need for expensive equipment.

## Materials and methods

**Figure 1.**
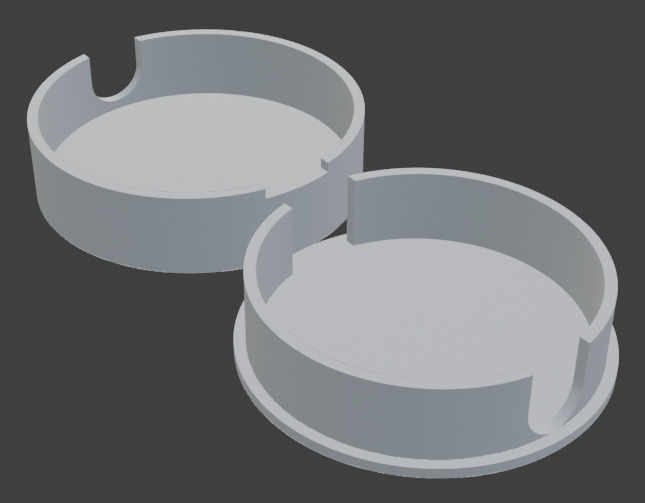
3D model of microphone case.

Materials:

- **Piezoelectric Discs**: These discs, available from electronic component suppliers, generate an electrical signal when subjected to mechanical vibrations. For our setup, we used a 27 mm white label disc with a resonant frequency of 4.6 (± 0.5) KHz and a resonant impedance of max 300 Ω;
- **Alligator clip**: Used to securely attach the piezo disc to substrates such as plant stems, minimising signal loss;
- **3D printed microphone housing**: A housing that contains the piezo element, the cables and the base of the alligator clip that serves to attach it to the substrate (Fig. 1, Suppl. material 1). To protect the device from electromagnetic interference, aluminium foil shields are placed on both sides of the piezo element and connected to the ground. A shielding aluminium foil and an alligator clip are attached to the base of the case using epoxy glue. After an additional insulating layer of epoxy adhesive is applied, the piezo element is attached. The remainder of the case is filled with epoxy adhesive, making the entire construction strong and moisture resistant. The 3D model of the sensor housing was created using the open source software Blender (Blender Online Community 2018);
- **Shielded Cable**: A shielded cable is used to connect the piezo disc to a recording device, preventing electromagnetic interference;
- **Recording Device with pre-amplifier**: A portable audio recorder with adequate sensitivity for vibration recording. Any digital recorder with a microphone input should be suitable. Three different recorders were used (see Recording setup). The best results were obtained using Zoom F6. Important for getting better recordings is using recorders with low preamp noise.

Construction:

- **Assembly**: The piezoelectric disc is connected to the coaxial cable by soldering the leads;
- **Attachment**: The piezoelectric sensor is attached to a substrate (e.g. plant stems, leaves or the ground) using adhesives or clips, ensuring close contact for optimal signal capture;
- **Recording Setup**: The output of the sensor is connected to an audio interface or portable recorder, which captures the vibrational signals.

Recording setup:

To record the signals of some of the insects (Hemiptera), test tubes were used in which plants and insects were placed (Fig. 2a). The tubes were clamped to the microphone with an alligator clip. Other insects (beetles, ants) were recorded by grasping the thorax with the tip of soft tweezers. The soft tweezers were, in turn, clamped with an alligator clip, with the pressure of the clamp being adjusted by the distance at which it was clamped - the closer to the tip of the tweezers the alligator clip was clamped, the stronger the insect was clamped (Fig. 2b).

In the setup shown in Fig. 2a, which has been tested in several Hemiptera for calling signals, the alligator clip is clamped on to the substrate, which can be either a plant stem or a test tube in which the plant is placed. In the experimental setup suitable for ants and beetles (Fig. 2b), the insects typically began to produce a distress signal spontaneously, as a result of stridulation. In contrast to the setup used in other studies, where the insect is held at a short distance from the microphone (e.g. Grasso et al. (2000), Ferreira et al. (2010), Peña Carrillo et al. (2021)), in our arrangement, the insect is connected to the contact sensor via the tweezers.

Signals were recorded using Tescam DR-60DMKII (96 kHz/24-bit), Zoom F3 (192 kHz/32-bit) and Zoom F6 (192 kHz/32-bit). All recorders have built-in preamps. Open source Audacity 3.6.1 software (Audacity Team 2024) was used for signal processing. For the preparation of oscillograms and sonograms, a Sonic Visualiser 5.0.1 was used (Cannam et al. 2010).

## Results and discussion

**Results**

Performance Evaluation:

- **Sensitivity**: The DIY piezoelectric contact microphone was capable of detecting vibrations in the typical range of insect signals (50–5000 Hz), similar to commercial devices. At the same time, since the frequency response of the piezo disc is known to depend on the environment, the mounting of the disc and many other parameters, the proposed sensor has its limitations and cannot be used for some types of biotremological studies. Nor can it be used to record vibrations at a specific point, as can be done with a laser vibrometer.
- **Cost**: The entire setup, including the piezo disc, cable and amplifier, costs less than $10, significantly more affordable than commercial solutions that often exceed $1000.
- **Durability**: The DIY sensor was tested in both laboratory and field conditions. It performed well under varying humidity and temperature conditions, demonstrating its suitability for field research.
- **Availability**: The developed setup uses a contact microphone based on a piezo disc, a modern component that is currently in production, unlike the gramophone cartridges that are largely obsolete and increasingly difficult to obtain.

Limitations:

- **Signal-to-Noise Ratio**: The DIY piezoelectric microphone showed slightly higher noise compared to commercial equipment. However, this can be mitigated through software post-processing or improved insulation.
- **Durability**: While the microphone performed well in short-term tests, long-term use in harsh environments could lead to wear and tear on the piezo discs or connections.

## Implementation

The study shows that a DIY piezoelectric contact microphone is a feasible, low-cost alternative for recording substrate-borne vibrations in insects. The low cost and ease of construction make this tool accessible for researchers with limited budgets or those working in the field.

In order to test the prepared sensor, we have recorded the signals of insects from different groups. Oscillograms, spectrograms and audio files of the recorded signals of *Messorwasmanni* Krausse, 1910 (Fig. 3, Suppl. material 2), *Myrmicaravasinii* Finzi, 1923 (Fig. 4, Suppl. material 3), *Manicarubida* (Latreille, 1802) (Fig. 5, Suppl. material 4), *Poneracoarctata* (Latreille, 1802) (Fig. 6, Suppl. material 5) (Hymenoptera, Formicidae), an ant nest beetle (*Paussusturcicus* I. Frivaldszky von Frivald, 1835) (Fig. 7, Suppl. material 6) (Coleoptera, Carabidae), a planthopper (*Orosangajaponica* (Melichar, 1898) (Fig. 8, Suppl. material 7) (Hemiptera, Ricaniidae) and a jumping plant louse (*Bactericeraperrisii* Puton, 1876) (Fig. 9, Suppl. material 8) (Hemiptera, Triozidae) are provided to demonstrate the effectiveness of the created equipment. An archive of the original unedited recordings presented in this article is provided (Suppl. material 9).

The recordings, processed with Audacity are available in the Supplementary files to this article. For some recordings, noise reduction was performed by sampling parts of the recording where there was no signal and suppressing the noise by 24-36 dB. For most recordings, a high-pass filter was applied at 50-100 Hz after ensuring that there was no signal to process at these frequencies. In some cases where the recording was too quiet, it was amplified. It is clear that the oscillograms and spectograms obtained are perfectly suitable for many types of analysis used in biotremology and also for taxonomic identification when acoustic libraries have been prepared for the group concerned, with the exception of the signals produced by the harvester ant *Messorwasmanni* and the psyllid *Bactericeraperrisii*. The recordings from the stridulation of the ants *Myrmicaravasinii*, *Manicarubida*, *Poneracoarctata*, the ant nest beetle *Paussusturcicus*, as well as and the male call song of the planthopper *Orosangajaponica*, are the first for these species. For *M.wasmanni*, only the high frequency signals were studied by Grasso et al. (2000) and no oscillogram was available, so it is not possible to compare the results, but the published oscillograms for *B.perrisii* (Tishechkin 2007) are very similar to those of the recordings with the proposed equipment.

## Re-use potential

The proposed equipment has some limitations compared to calibrated sensor instruments used in biotremological studies. This is due to the frequency response of the piezo discs being dependent on the DYI sensor assembly. Meanwhile, the substrate on which a vibration-generating insect resides is an essential component of the vibration transmission channel. The frequency responses of most substrates, such as plant stems, exhibit significant variability and non-linearity (Michelsen et al. 1982). Consequently, the inherent non-linearity of the sensor response is likely to introduce minimal additional distortion to the recorded signal and can, therefore, be considered negligible. Nevertheless, the sensor can be used to record biotremological data, allowing comparative analysis of the signal structure and a range of parameters. The principal advantage of the proposed DYI biotremological sensor is its accessibility: it can be reproduced in a laboratory or at home by any individual, including those without any specialised knowledge of electronics.

## Conclusions

The research presented will democratise biotremological methods for the needs of integrative taxonomy and behavioural ecology. The DIY piezoelectric contact microphone developed in this study is a practical, low-cost tool for recording insect vibrations from both professional and citizen scientists. It offers comparable performance to commercial sensors at a fraction of their cost, making biotremology research more accessible to a wider audience. Future refinements could include better noise insulation and more robust construction for long-term use in various environments.

## Supplementary Material

8EA94408-8110-574A-AAF1-7DDFB559B50B 10.3897/BDJ.13.e143481.suppl1 Supplementary material 1 3D printed housing of piezoelement and alligator clip Data type stl 3D model Brief description STL model housing for mounting a piezo disc and crocodile clip. The housing is for use with a 28 mm diameter piezo disc. File: oo_1167425.stl https://binary.pensoft.net/file/1167425 Ilia Gjonov, Albena Lapeva-Gjonova, Monika Pramatarova

591E2FD6-44AB-5F9F-A35C-A8EA73C06779 10.3897/BDJ.13.e143481.suppl2 Supplementary material 2 Distress signal of Messorwasmanni Data type sound file File: oo_1180007.wav https://binary.pensoft.net/file/1180007 Ilia Gjonov, Albena Lapeva-Gjonova, Monika Pramatarova

86908E64-1E6D-5B8D-AA2F-F820D839CE2F 10.3897/BDJ.13.e143481.suppl3 Supplementary material 3 Distress signal of Myrmicaravasinii Data type sound file File: oo_1223518.wav https://binary.pensoft.net/file/1223518 Ilia Gjonov, Albena Lapeva-Gjonova, Monika Pramatarova

D8E0BA84-2BEB-5D3B-9959-D49A2479DA7A 10.3897/BDJ.13.e143481.suppl4 Supplementary material 4 Distress signal of Manicarubida Data type sound file File: oo_1223517.wav https://binary.pensoft.net/file/1223517 Ilia Gjonov, Albena Lapeva-Gjonova, Monika Pramatarova

D189D8EA-1C1D-5B7B-A6D4-C029EF9F6849 10.3897/BDJ.13.e143481.suppl5 Supplementary material 5 Distress signal of Poneracoarctata Data type sound file File: oo_1223543.wav https://binary.pensoft.net/file/1223543 Ilia Gjonov, Albena Lapeva-Gjonova, Monika Pramatarova

D74C233A-6C67-5190-8FD9-2E7BB4323403 10.3897/BDJ.13.e143481.suppl6 Supplementary material 6 Distress signal of Paussusturcicus Data type sound file File: oo_1180015.wav https://binary.pensoft.net/file/1180015 Ilia Gjonov, Albena Lapeva-Gjonova, Monika Pramatarova

50FBE803-864F-5B31-A482-32964E07D393 10.3897/BDJ.13.e143481.suppl7 Supplementary material 7 Male calling song of Orosangajaponica Data type sound file File: oo_1180016.wav https://binary.pensoft.net/file/1180016 Ilia Gjonov, Albena Lapeva-Gjonova, Monika Pramatarova

5C6367EF-8EC8-59CE-81F8-C474A682DC4E 10.3897/BDJ.13.e143481.suppl8 Supplementary material 8 Male calling song of Bactericeraperrisii Data type sound file File: oo_1180017.wav https://binary.pensoft.net/file/1180017 Ilia Gjonov, Albena Lapeva-Gjonova, Monika Pramatarova

217E2D45-7358-5C4C-A7CB-B7B426806CB7 10.3897/BDJ.13.e143481.suppl9 Supplementary material 9 Raw recordings Data type sound recordings Brief description An archive of the original unedited recordings presented in this article. File: oo_1224091.zip https://binary.pensoft.net/file/1224091 Ilia Gjonov, Albena Lapeva-Gjonova, Monika Pramatarova

## Figures and Tables

**Figure 2a.**
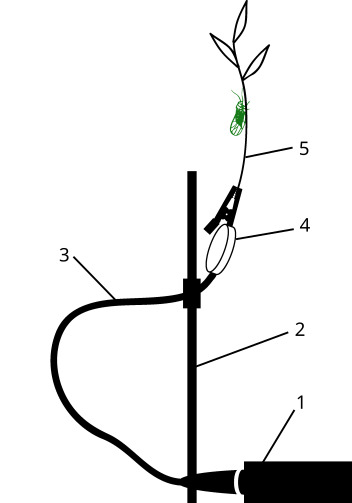
calling signals in Hemiptera;

**Figure 2b.**
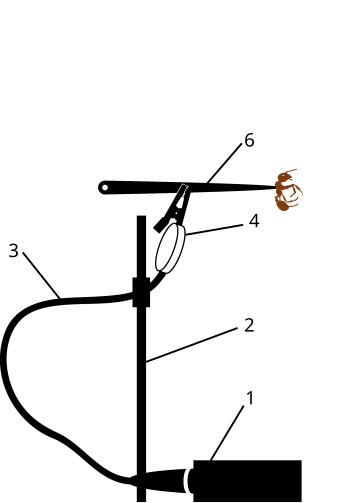
signals from stridulation in Formicidae and Coleoptera.

**Figure 3a.**
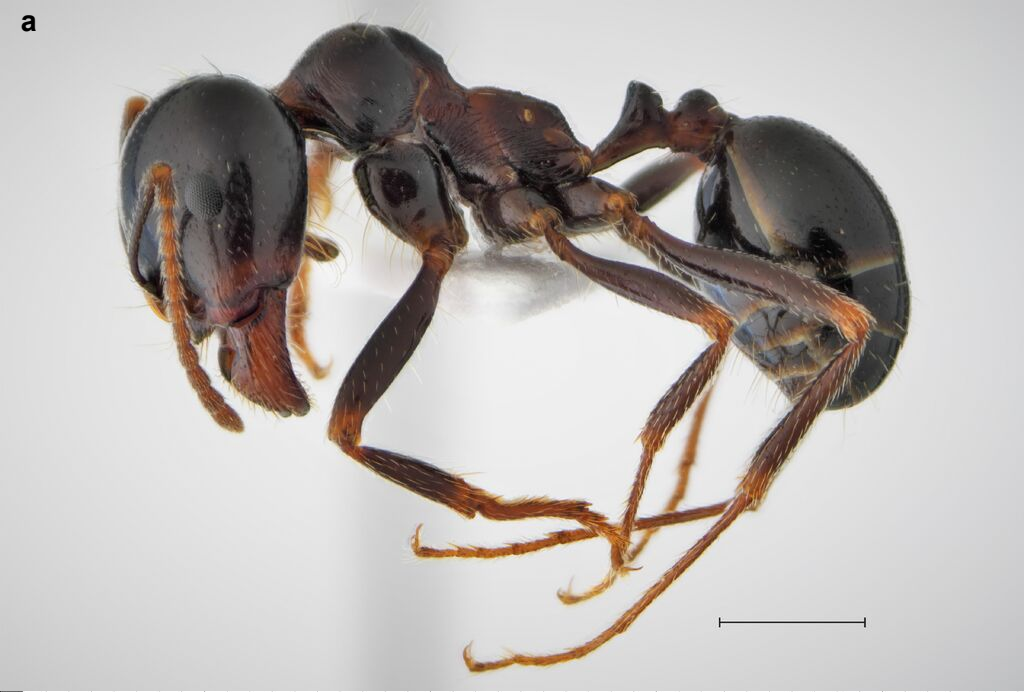
Habitus, scale: 1 mm;

**Figure 3b.**
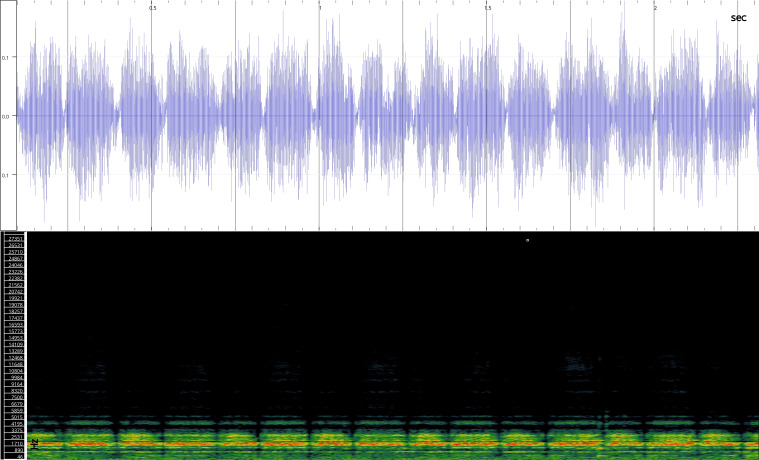
Oscillogram and spectrogram.

**Figure 4a.**
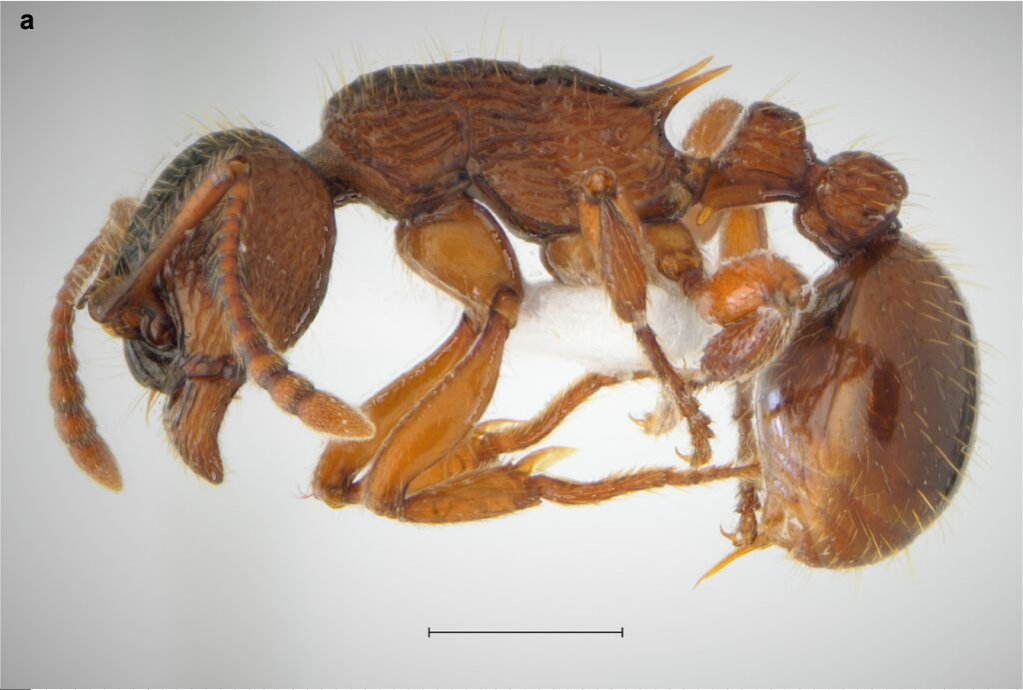
Habitus, scale: 1 mm;

**Figure 4b.**
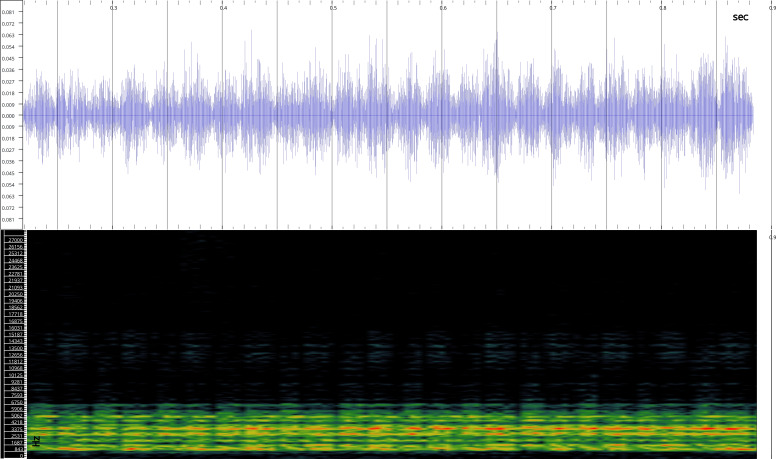
Oscillogram and spectrogram.

**Figure 5a.**
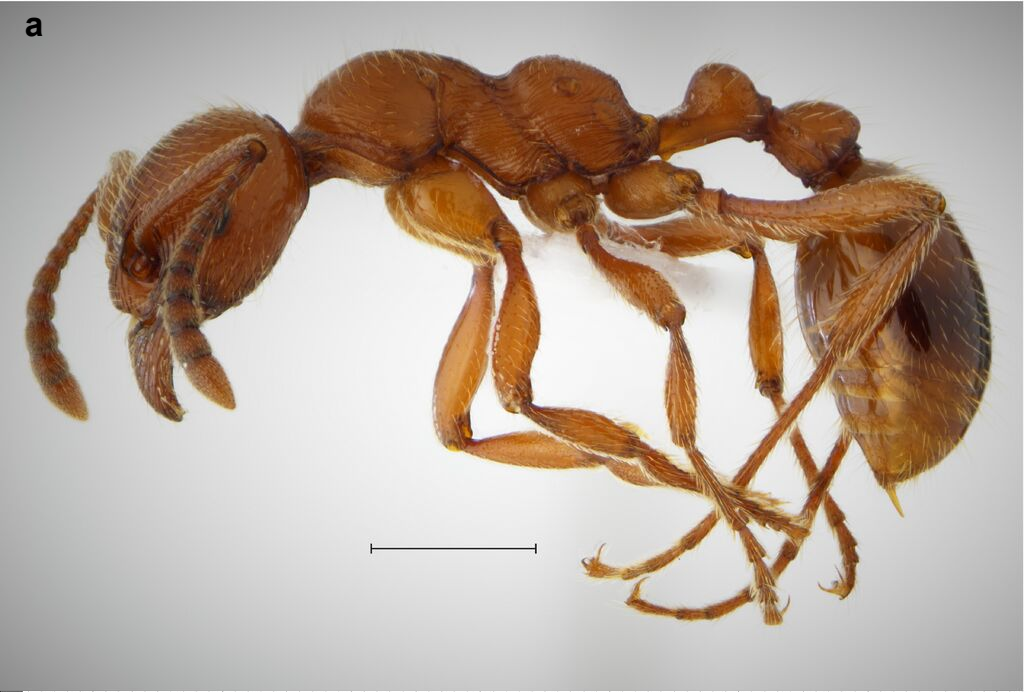
Habitus, scale: 1 mm;

**Figure 5b.**
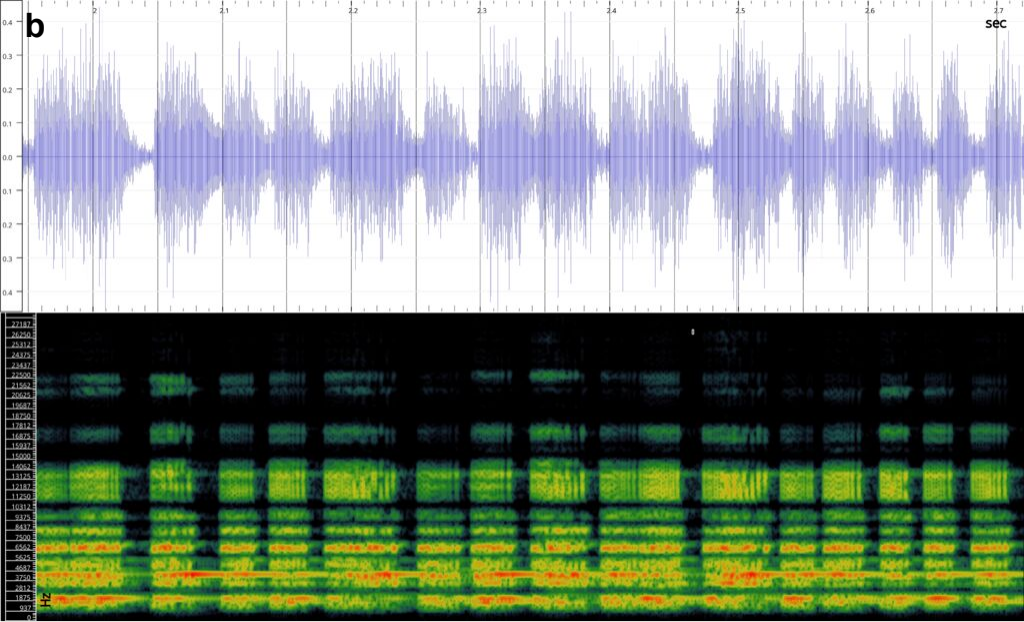
Oscillogram and spectrogram.

**Figure 6a.**
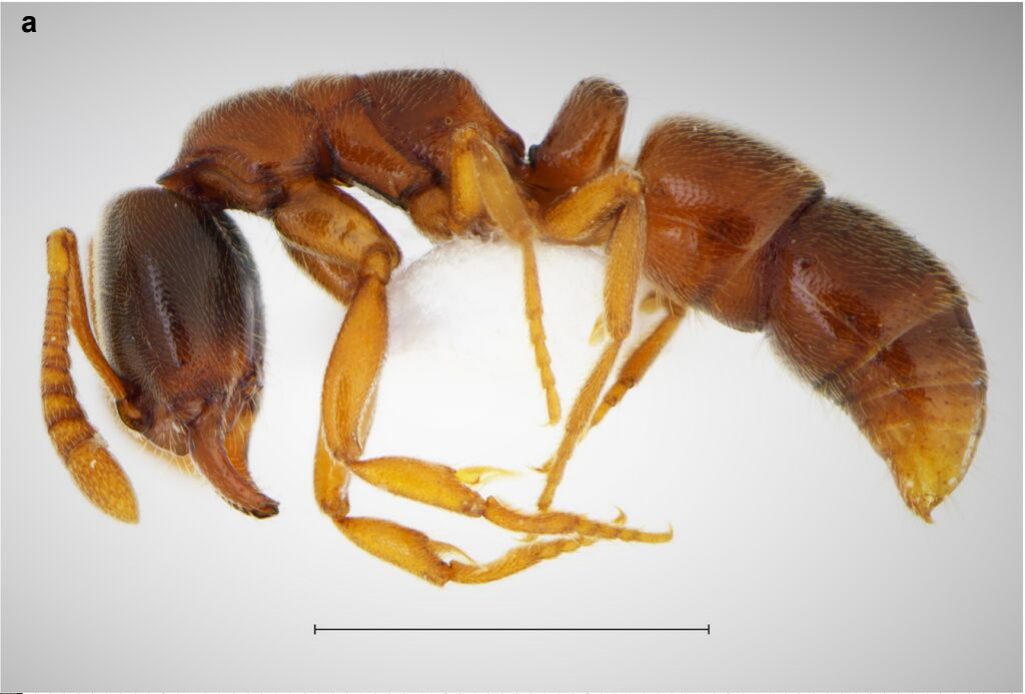
Habitus, scale: 1 mm;

**Figure 6b.**
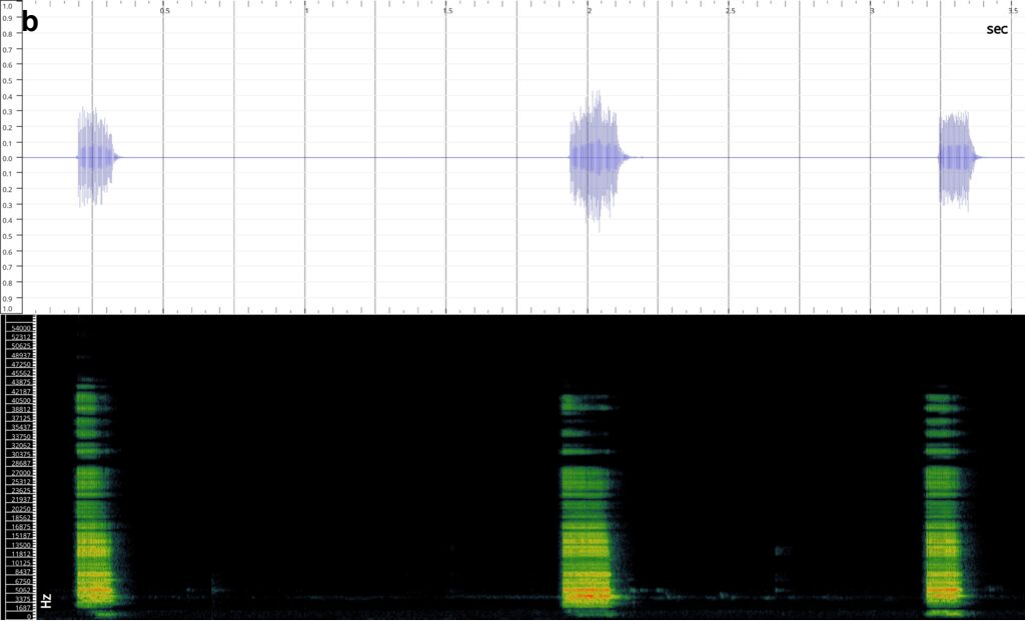
Oscillogram and spectrogram.

**Figure 7a.**
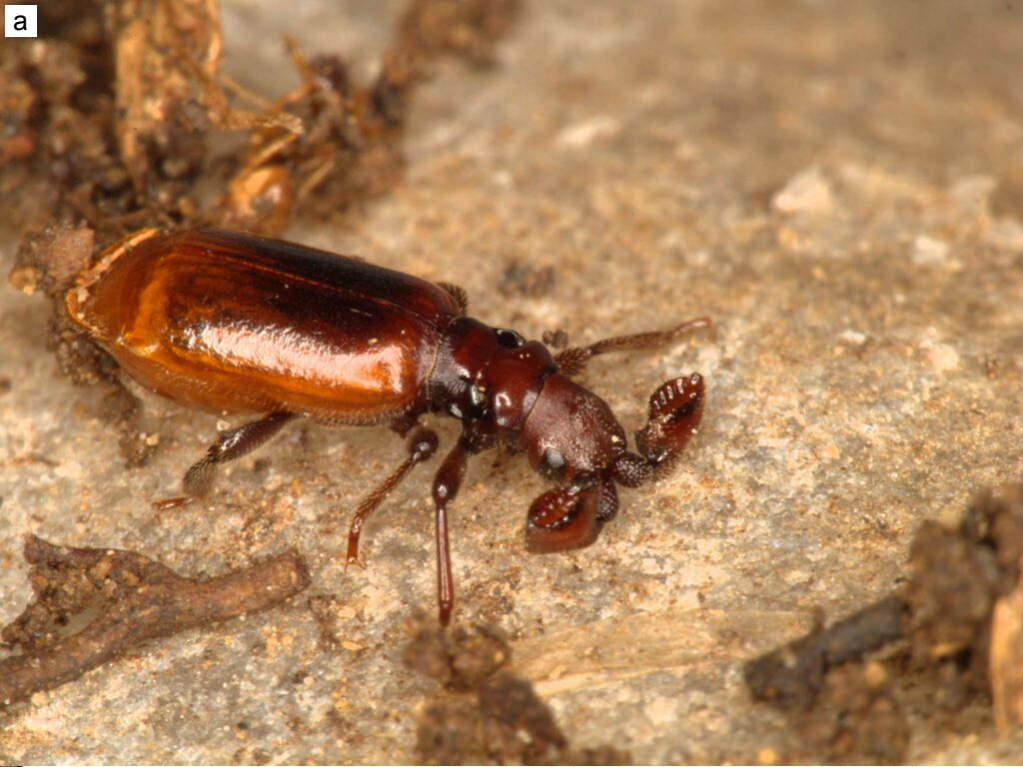
Habitus;

**Figure 7b.**
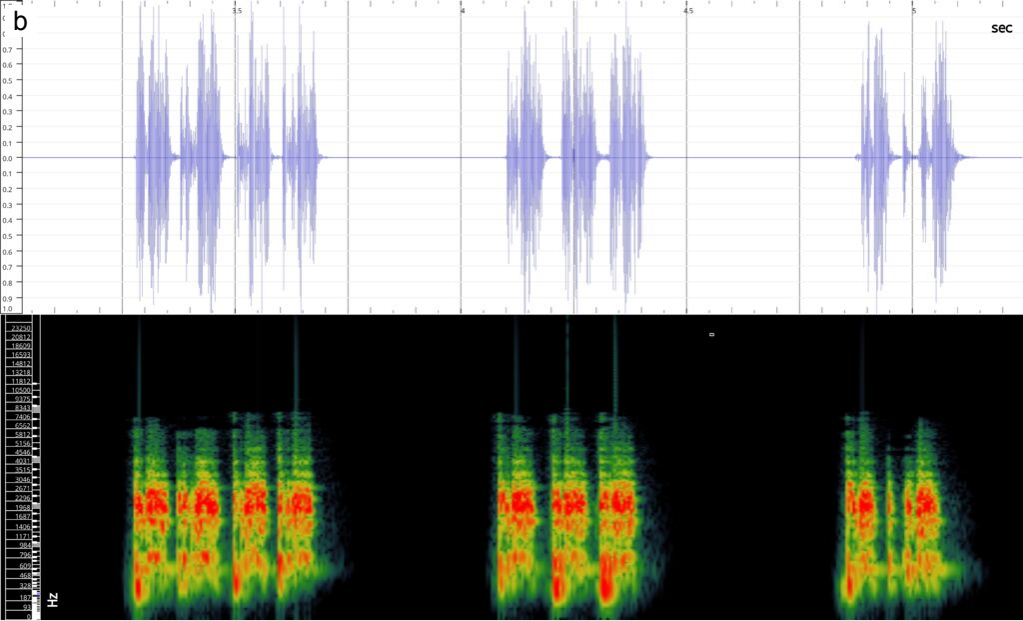
Oscillogram and spectrogram.

**Figure 8a.**
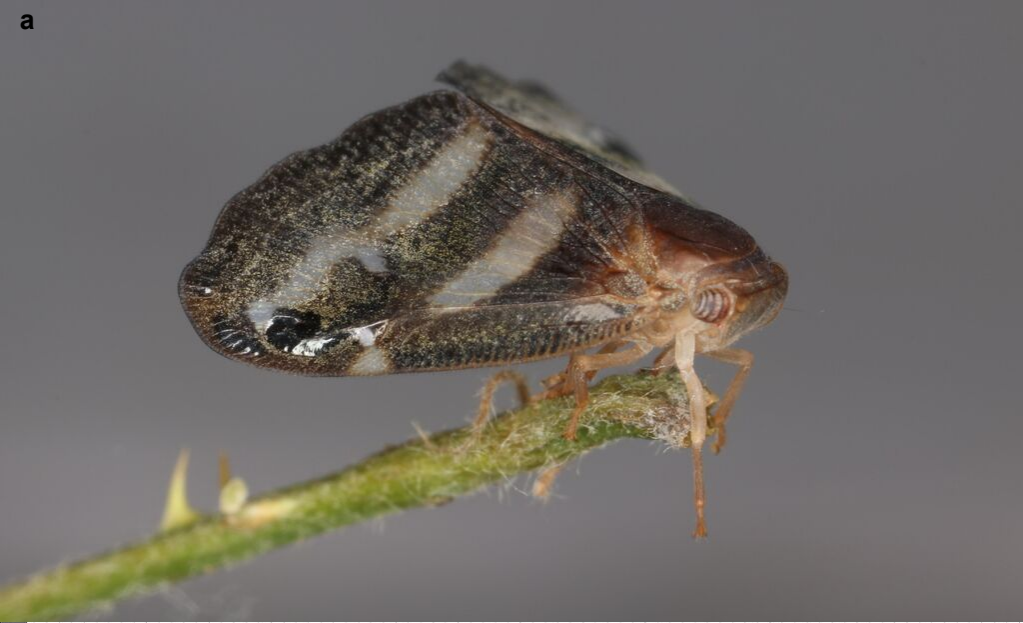
Habitus;

**Figure 8b.**
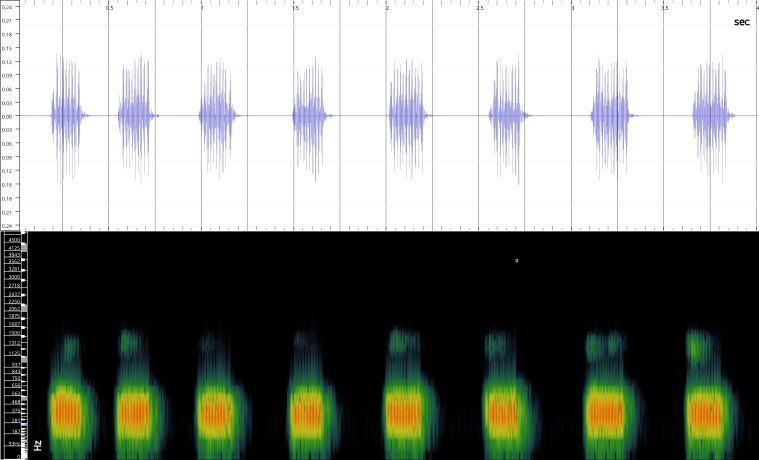
Oscillogram and spectrogram.

**Figure 9a.**
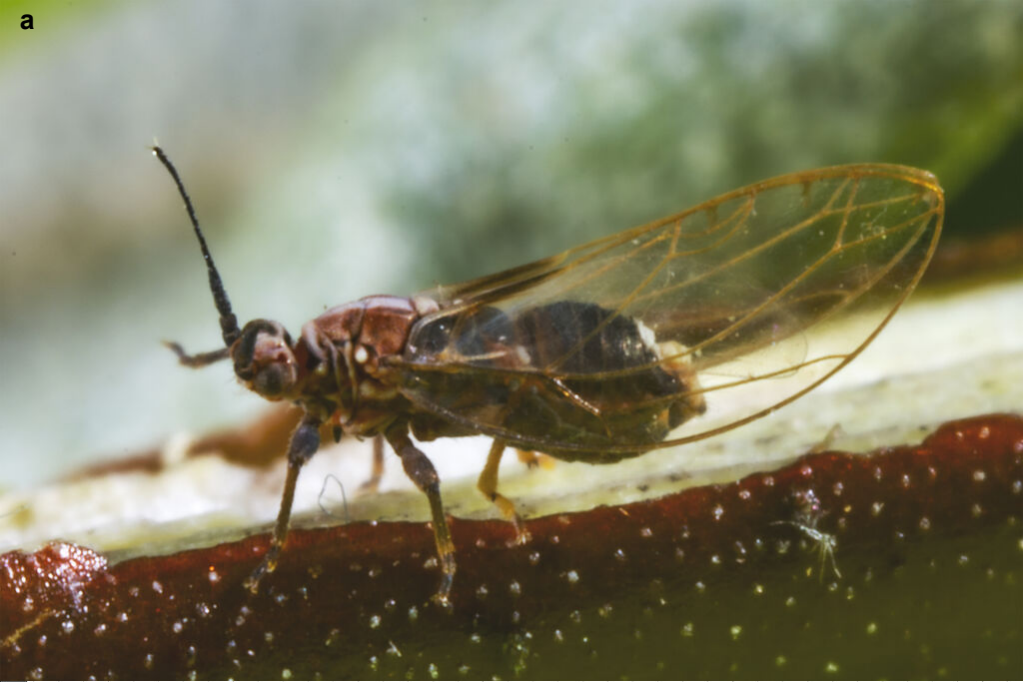
Habitus;

**Figure 9b.**
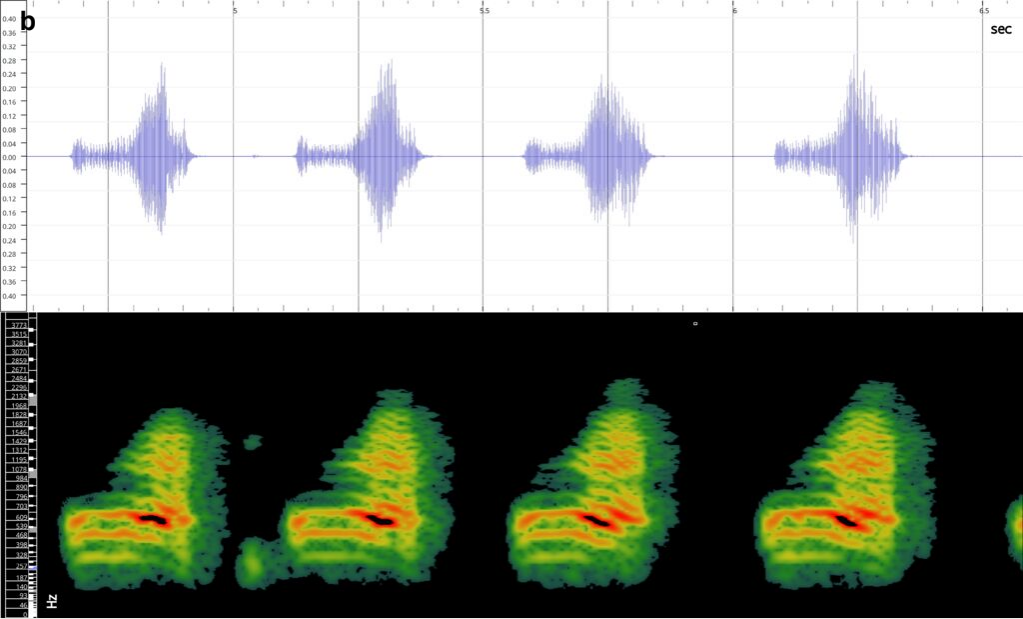
Oscillogram and spectrogram.
